# Comparing Longitudinal Changes in Physical Activity and Fear of Falling in Non-Fallers, Fallers, and Injurious Fallers

**DOI:** 10.1093/geroni/igaa057.2781

**Published:** 2020-12-16

**Authors:** Jian-Yu E, Jennifer Schrack, Aleksandra Mihailovic, Tianjing Li, David Friedman, Sheila West, Laura Gitlin, Pradeep Ramulu

**Affiliations:** 1 Johns hopkins Bloomberg School of Public Health, Baltimore, Maryland, United States; 2 Johns Hopkins Bloomberg School of Public Health, Baltimore, Maryland, United States; 3 Johns Hopkins University/Wilmer Eye Institute, Baltimore, Maryland, United States; 4 University of Colorado, Arora, Colorado, United States; 5 Massachusetts Eye and Ear, Harvard Medical School, Boston, Massachusetts, United States; 6 Johns Hopkins School of Medicine, Baltimore, Maryland, United States; 7 Drexel University, Philadelphia, Pennsylvania, United States

## Abstract

Older adults with visual impairments experience a higher risk of falling, and are more vulnerable to associated adverse health consequences than those with normal vision. We investigated the onset and magnitude of declines in physical activity (PA), and corresponding changes in self-reported fear of falling (FoF) in 234 visually impaired persons (mean age=69.8, 48.7% women) over three years. PA was measured using the Actical hip-worn accelerometer and falls were reported using a calendar. In fully adjusted linear models, PA declined 426 steps/year (p<0.01) and 15.1 active minutes/year (p<0.01) among injurious fallers compared to non-fallers; PA did not change among non-injurious fallers. No longitudinal declines in FoF scores were observed. Among visually impaired older adults, an injurious fall contributed to subsequent declines in activity, although FoF remained unchanged. Further longitudinal research is warranted to better understand how different groups respond to falls, either by behavioral changes and/or changes in FoF.

